# Combination autologous stem cell transplantation with chimeric antigen receptor T-cell therapy for refractory/relapsed B-cell lymphoma: a single-arm clinical study

**DOI:** 10.3389/fimmu.2025.1532460

**Published:** 2025-02-26

**Authors:** Danyang Li, Rui Liu, Zhonghua Fu, Fan Yang, Lixia Ma, Yuelu Guo, Miaomiao Cao, Yang Lei, Yimeng Dou, Xuenan Zhang, Yan Gao, Bian Wei, Biping Deng, Xiaoyan Ke, Kai Hu

**Affiliations:** ^1^ Department of Lymphoma and Myeloma Research Center, Beijing GoBroad Hospital, Beijing, China; ^2^ Department of Hematology, Peking University Third Hospital, Beijing, China

**Keywords:** B-NHL, CAR-T, ASCT, combination therapy, refractory/relapsed B-cell lymphoma

## Abstract

Autologous stem cell transplantation (ASCT) and chimeric antigen receptor T-cells (CAR-T) have been used as consolidation therapies for patients with refractory/relapsed B cell non-Hodgkin’s lymphoma (R/R B-NHL) in remission after second-line chemotherapy or salvage therapy. However, patients with different pathological subtypes and remission states may benefit differently from ASCT or CAR-T cell therapy. Furthermore, consolidation treatment involving ASCT or CAR-T cells still poses a significant risk of disease relapse. We conducted a retrospective, single-arm study of 47 patients with R/R B-NHL, and found that the combination of ASCT and CAR-T therapy improved the 3-year progression-free survival (PFS) and overall survival (OS) rates to 66.04% (95%CI: 48.311-78.928) and 72.442% (95%CI: 53.46-84.708) respectively. Furthermore, the combination therapy has no serious adverse events. Thus, ASCT combined with CAR-T cell therapy is effective against multiple subtypes of R/R B-NHL, and can effectively prolong the long-term survival of patients.

## Introduction

Non-Hodgkins lymphoma (NHL) is the most common hematological malignancy (1), and B-cell lymphomas account for about 85% of all NHL cases (2). B-cell NHL can be classified into various pathological subtypes, from relatively indolent lymphomas to highly aggressive lymphomas (3). Standard treatment options such as R-CHOP (rituximab in combination with chemotherapy) have been effective against diffuse large B-cell lymphoma (DLBCL), the most common form of B-NHL, with about 60-70% of the patients achieving complete remission and long-term survival (4, 5). For patients with relapsed/refractory DLBCL (R/R DLBCL) however, the current salvage chemotherapy regimen combined with autologous stem cell transplantation (ASCT) consolidation therapy has a cure rate of only 10% (6). Chimeric antigen receptor T cell (CAR-T) therapy is used in patients who relapse after second-line therapy, although there is a risk of relapse after cure (7, 8).

ASCT is a standard treatment for R/R B-NHL that is still sensitive to chemotherapy. Studies have shown that auto-HCT can significantly improve clinical outcomes and survival in patients who achieve complete response (CR) after the chemotherapy regimen. However, ASCT alone does not fully eradicate minimal residual disease (MRD) (9), thereby posing a risk of relapse in certain non-responsive patients who exhibit sub-optimal clinical outcomes after salvage chemotherapies. Consequently, the anticipated long-term progression-free survival (PFS) rate of patients undergoing ASCT ranges from 10% to 30% (10, 12). CAR-T cell therapy involves genetically modifying autologous T cells to recognize and attack tumor cells, and is particularly effective in patients who are resistant to conventional treatments (13, 14). However, CAR-T cell monotherapy faces challenges such as T-cell exhaustion and uncertainties regarding long-term efficacy (15). Although CAR-T cell infusion is widely used for the treatment of R/R B-NHL at present (16), and has achieved some efficacy as a consolidation therapy, there is still a risk of recurrence (14, 17, 18). Overall, the anti-tumor effects of ASCT and the specific targeting ability of CAR-T cells can synergistically increase the rate of CR and extend the PFS of cancer patients. Nevertheless, clinicians need to consider the indications, efficacy, and potential side effects of each approach in individual patients in order to develop personalized treatment plans. In this study, we evaluated the clinical outcomes of combining ASCT and CAR-T cell infusion as the consolidation therapy for R/R B-NHL patients. Our findings suggest that this combination therapy can improve the long-term survival and quality of life of patients.

## Methods

### Patients

Patient diagnosed with R/R B-NHL who underwent consecutive ASCT and CAR-T cell therapy between October 2019 and November 2023 were enrolled. Diagnosis was established through histopathological evaluation and imaging, and the B-NHL subtype was determined according to the criteria of World Health Organization 2016 classification of tumors of hematopoietic and lymphoid tissues. The prognostic profiles of the patients were assessed using the International Prognostic Index (IPI). Clinical staging adhered to the Ann Arbor staging system, along with an ECOG performance status of 0-2, to ensure accurate stratification and informed treatment decisions. The study complied with the principles of the Declaration of Helsinki and was approved by the Institutional Ethics Committee at Beijing Gobroad Hospital. All participants provided written informed consent.

Relapse was defined as the emergence of any novel lesion, or a 50% or greater increase in the size of previous lesions, subsequent to achieving a CR (19). Refractory disease was characterized by the failure to attain at least a partial response (PR) following chemotherapy, either after more than four cycles of first-line therapy or more than two cycles of subsequent lines of therapy, or by the relapse of disease within one-year post-ASCT (20, 21).

### Inclusion and exclusion criteria

**Figure 1:**
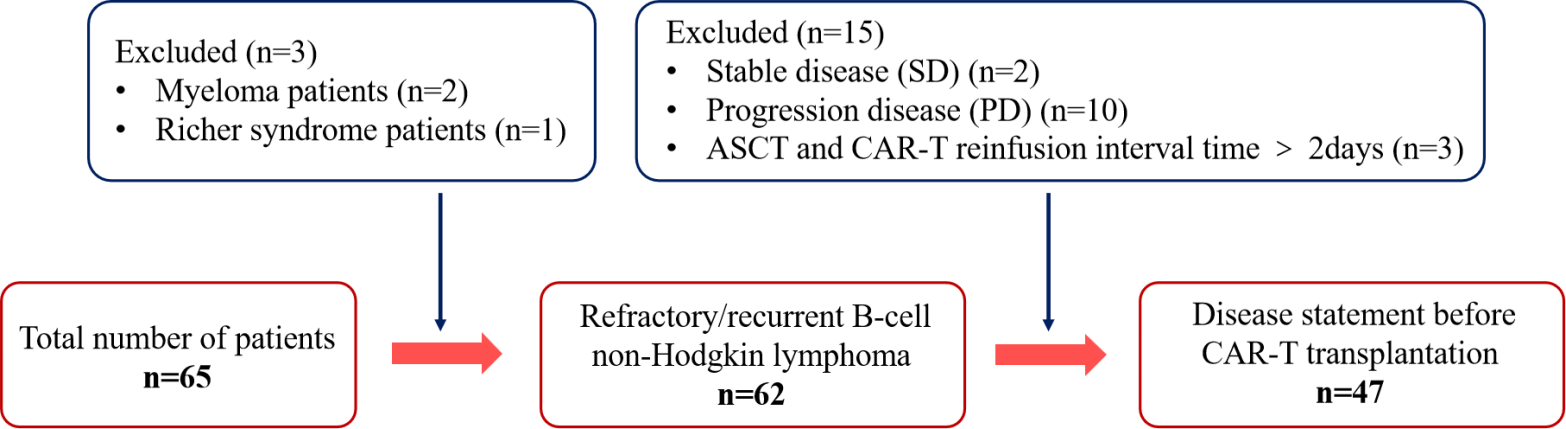
Flow diagram of patient selection. Status of enrolled patients in the autologous stem cell transplantation combined with chimeric antigen receptor T-cell therapy.

The inclusion criteria for the patients were as follows: 1) age ranging from 16-69 years, 2) confirmed diagnosis of NHL, and 3) CAR-T cell re-infusion received within two days of ASCT. Patients who received CAR-T cell re-infusion more than 2 days after ASCT were excluded (n=3). In addition, patients with myeloma (n=2) and Richer syndrome (n=1) were also excluded to avoid any confusion due to the distinct pathological features and treatment response of these diseases relative to B-NHL. Finally, patients with stable disease (SD) (n=2) or progression disease (PD) (n=10) before CAR-T cells transplantation were excluded, as lack of a sufficient response before CAR-T cell therapy may have affected the evaluation of consolidation therapy. Finally, 47 patients were included in the study (**Figure 1**).

### Study design

**Figure 2:**
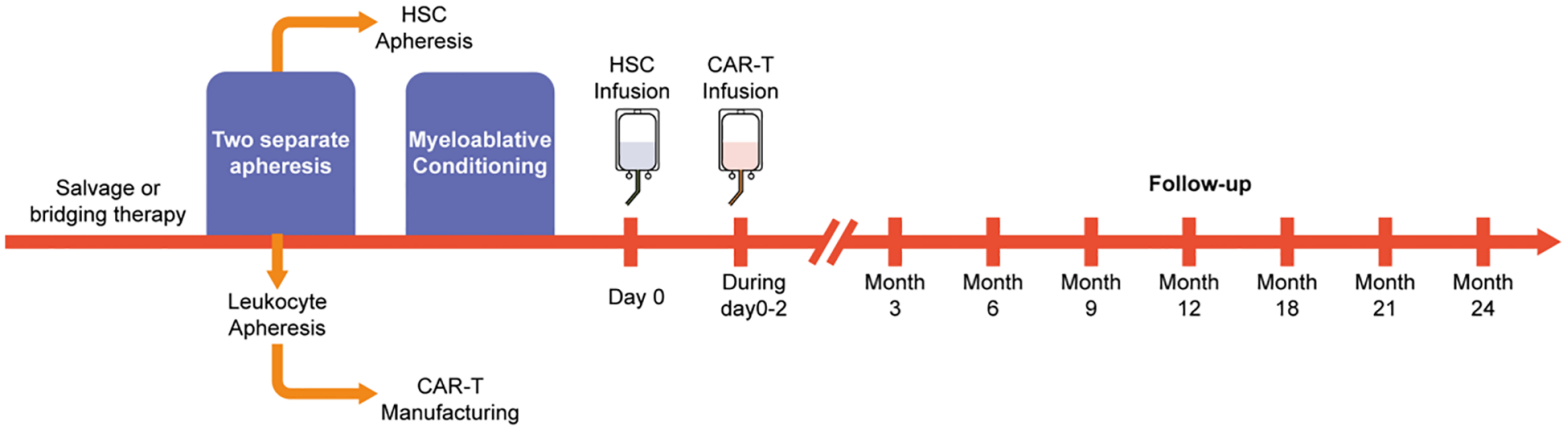
Flow diagrams of the treatment. The figure illustrates the entire process of ASCT combined with CAR-T treatment.

The combination therapy regimen is outlined in **Figure 2**. Granulocyte colony-stimulating factor-primed autologous stem cells and peripheral lymphocytes (~50 ml) were harvested from each patient through distinct apheresis procedures. CD19-41BB-CAR-T cells were generated from CD3+ T cells isolated from the apheresed lymphocytes. All patients received high-dose chemotherapy before the infusion of autologous stem cells. The pre-conditioning regimens were designed according the disease subtype. For instance, standard BEAM (bis-carmustine, etoposide, cytarabine, and melphalan) and TT-BuCy (Thiotepa Semustine, Cytarabine, Busulfan) was used for patients with central system invasion. The dose of CD34+ hematopoietic stem cells (HSCs) ranged from 0.4 to 9.5*10^6^/kg. CAR-T cells were transfused within two days after transfusion of CD34+ HSCs. Based on prior clinical expertise with B-NHL patients, the dose of CAR-T cells ranged from 0.4 to 7.5*10^6^/kg.

### Measurement of clinical endpoints

The primary objective of the trial was to assess the safety and tolerability of the combination therapy. Secondary objectives were to evaluate treatment efficacy, PFS, and overall survival (OS). The treatment responses were evaluated using computed tomography or positron emission tomography in accordance with the 2014 Lugano Recommendations for Lymphoma (22). Bone marrow biopsies were taken from patients exhibiting bone marrow infiltration. The overall response rate (ORR) was calculated as the sum of CR and PR. PFS was defined as the period from the initiation of CAR-T therapy or ASCT until disease progression, relapse, or mortality, whichever transpired first. OS was measured from the onset of CAR-T therapy or ASCT until death from any cause. Adverse events (AEs) were graded by the Common Terminology Criteria for Adverse Events version 5.0. CAR-T cell therapy-related AEs, including neurotoxicity and cytokine release syndrome (CRS), were assessed using the Penn scale (23). Deaths and their potential causes were recorded, and therapy-related deaths were analyzed in detail.

### Statistical analysis

The demographic and other baseline data have been presented as frequencies and percentages. The probabilities of OS and PFS were calculated by the Kaplan-Meier method and compared using log-rank test. The 95% confidence intervals (CI) for survival were calculated using the GraphPad Prism V.9.0 software. SPSS version 26.0 and GraphPad Prism version 9.0 were used for data analysis. A two-sided p-value < 0.05 was considered statistically significant.

## Results

### Patient characteristics

Between October 2019 and November 2023, a total of 65 patients underwent CAR-T therapy after ASCT, of which 47 patients fulfilled the inclusion criteria and were included in the study. The baseline characteristics of the patients are summarized in **Table 1**. The median age of the patients was 38 years (16 to 69 years), and 59.6% (n=28) of the patients were males. The pathological subtypes of R/R B-NHL in this cohort were DLBCL, primary mediastinal lymphoma (PML), primary central nervous system lymphoma (PCNSL) and high-grade B-cell lymphoma (HGBL) with MYC and/or BCL2/BCL6 rearrangement. All patients who were included in the study had achieved CR or PR. The interval between ASCT and CAR-T transfusion was 0, 1, and 2 days in 2, 25, and 20 patients respectively (**Table 1**).

**Table 1:** Characteristics of patients at baseline.

Characteristic/Variable	Patients (N=47)
Age	
Median age(range)-year	38 (16-69)
Sex	
Male-no.(%)	28 (59.6)
Female-no.(%)	19 (40.4)
ECOG performance status-no.(%)	
0-1	36 (76.6)
≥2	11 (23.4)
Disease status at study entry-no.(%)	
Refractory disease	14 (29.8)
Relapsed disease	33 (70.2)
Treatment history and early efficacy	
Early therapy failure in 12months-no.(%)	25 (75.8)
Prior lines of therapy at study, median (range)	4 (2 - 8)
Disease type-no.(%)	
BL	2 (4.3)
DLBCL	22 (46.8)
DLBCL secondary central nervous system	4 (8.5)
HGBL	5 (10.6)
HGBL secondary central nervous system	2 (4.3)
PCNSL	3 (6.4)
PMBL	7 (14.9)
PMBL secondary central nervous system	2 (4.3)
Prior disease status-no.(%)	
CR	27 (57.4)
CMR	6 (12.8)
CRu	2 (4.3)
PR	12 (25.5)
Time interval between transplantation and infusion-day	
0	2 (4.3)
1	25 (53.2)
2	20 (42.6)

BL, Burkitt lymphoma; DLBCL, diffuse large B cell; MCL, mantle cell lymphoma, HGBL, High-grade B-cell lymphoma; PCNSL, Primary central nervous system lymphoma; PMBL, primary mediastinal large B-cell lymphoma; PBL, plasmablastic lymphoma; CR, complete response; CMR, complete metabolic response; CRu, complete response unconfirmed; PR, partial response.

### Efficacy of combination therapy

**Figure 3:**
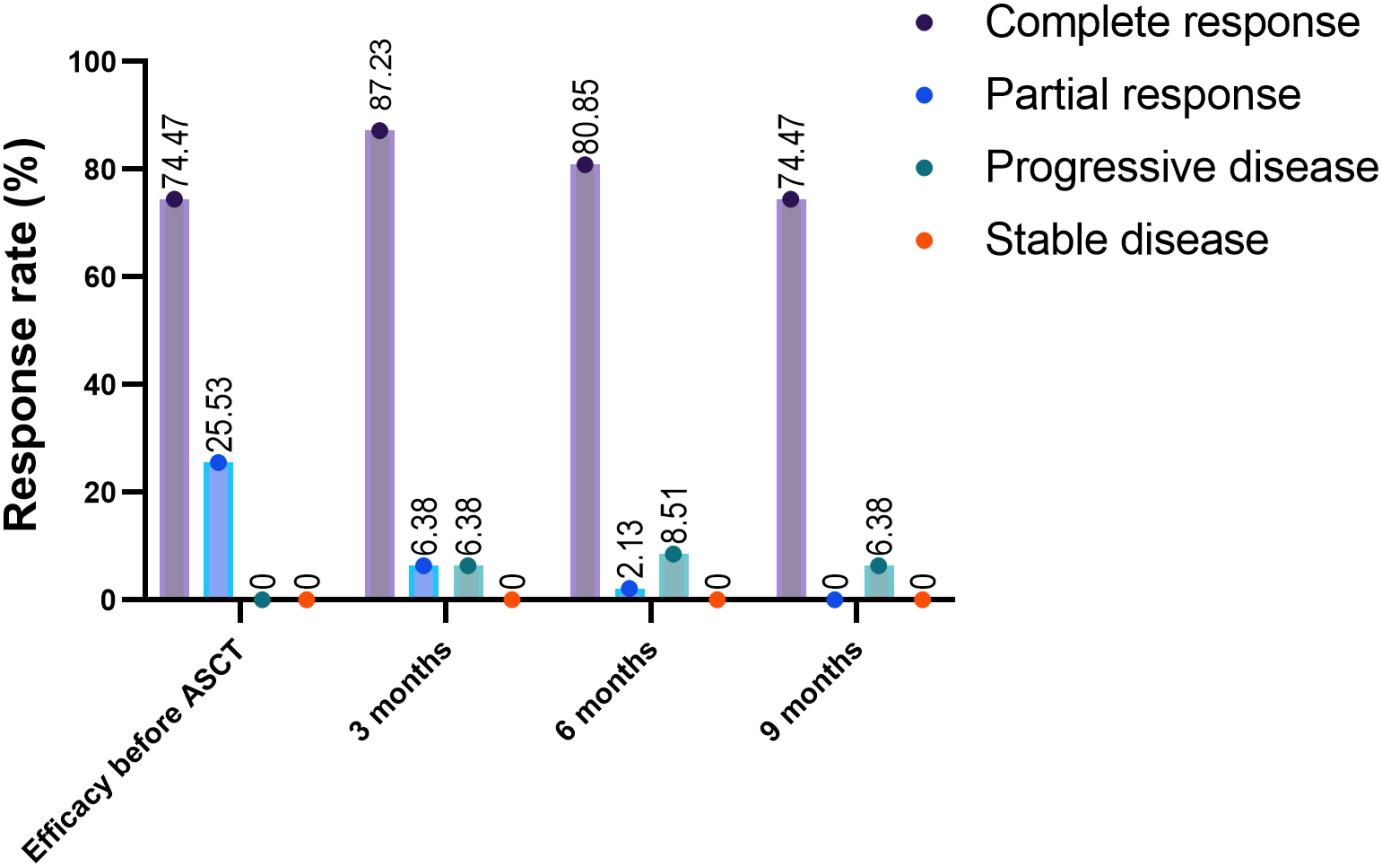
Response rates after ASCT and CAR-T combination therapy. The response rates before and 3 and 6 months after undergoing ASCT and CAR-T cell combination therapy. ASCT, autologous stem cell transplantation; ORR, overall response rate.

**Figure 4:**
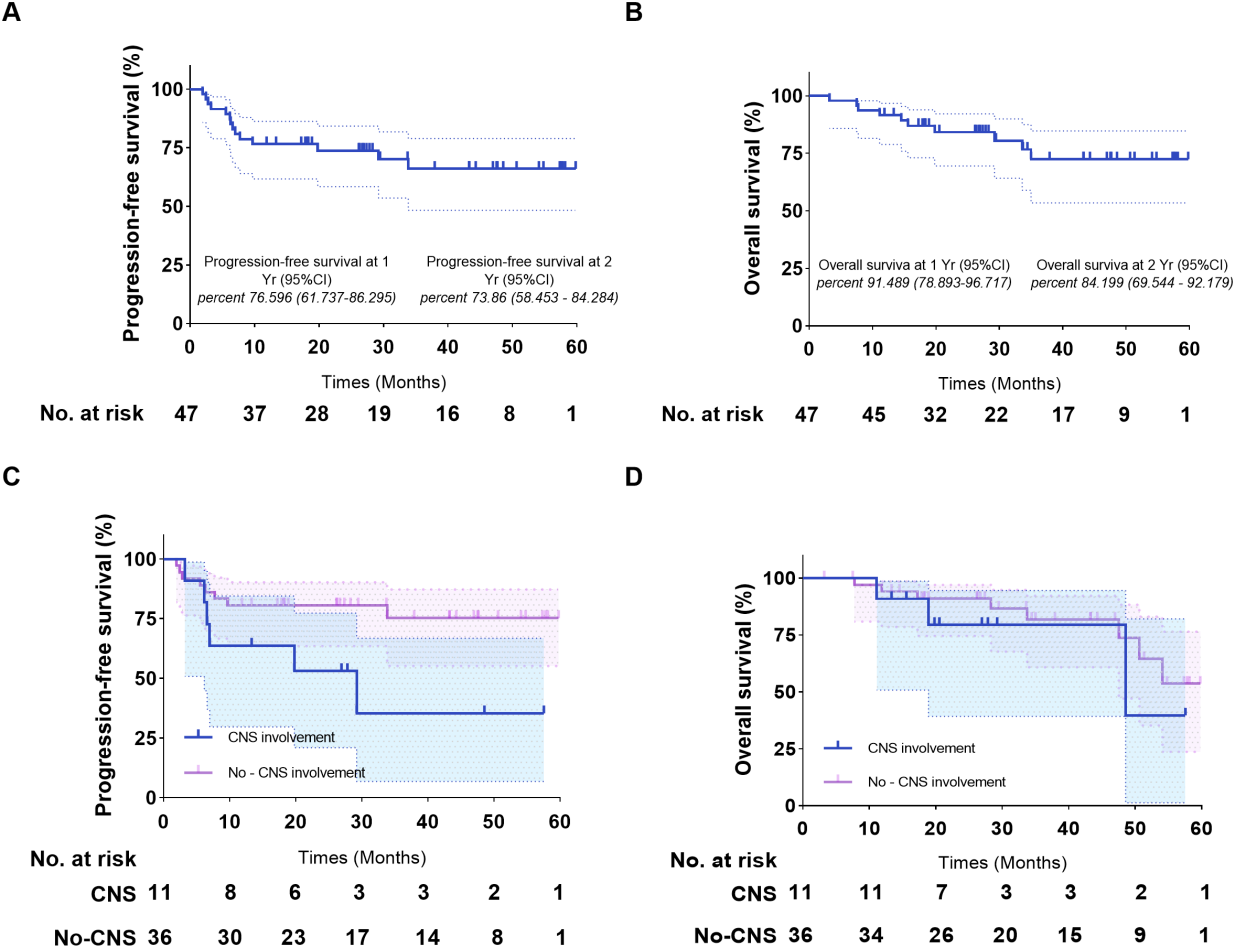
Kaplan-Meier estimates of the progression-free survival and overall survival of patients who underwent ASCT and CAR-T cell infusion. **(A)** Progression-free survival of the entire cohort. **(B)** Overall survival of the entire cohort. **(C)** Progression-free survival of patients with/without CNS involvement. **(D)** Overall survival of patients with/without CNS involvement. ASCT, autologous stem cell transplantation.

As of October 15, 2024, the median follow-up duration from ASCT to the data cut-off date was 28.3 months. CAR-T levels peaked in the peripheral blood at a median of 11 days after infusion (range 6-60 days) and lasted for a median duration of 21 days (range 4-90 days). The median maximum expansion of the CAR-T cells was 55.15*10^6^ cells/L (range 0.127*10_6_, 1970*10_6_). Furthermore, the median time to the engraftment of neutrophils and platelets after hematopoietic stem cell transplantation was 14 days (range 10-62 days) and 15 days (range 15-52 days) respectively. All patients responded to pretreatment, with CR of 72% and PR of 28%. As shown in **Figure 3**, 85.42%, 79.17%, and 72.92% of the patients had an ongoing response at respectively 3, 6, and 9 months after combination therapy (**Figure 3**). The 1-, 2-, and 3-year PFS rates were 76.596% (95% CI 61.737% to 86.295%), 72.321% (95% CI 57.028% to 82.964%), and 64.664% (95% CI 47.228% to 77.619%) respectively, and the 1-, 2-, and 3-year OS rates were 91.489% (95% CI 78.893% to 96.717%), 73.86% (95% CI 58.453% to 84.284%), and 66.04% (95% CI 48.311% to 78.928%) respectively (**Figures 4A, B**). The number of surviving and deceased patients at these specified time points are summarized in **Table 2**. We also classified the patients according to central nervous system (CNS) involvement to determine a potential impact on therapeutic efficacy. The results have been summarized in **Table 3** and **Figures 4C, D**.

**Table 2:** Clinical response in combination therapy.

	Status		%(95% CI)
	Live	Dead	
OS				
1-year	44	3	91.489 (78.893-96.717)
2-year	42	5	73.86 (58.453-84.284)
3-year	40	7	66.04 (48.311-78.928)
PFS				
1-year	36	11	76.596 (61.737-86.295)
2-year	35	12	72.321 (57.028-82.964)
3-year	33	14	64.664 (47.228-77.619)

**Table 3:** Clinical response in patients with/without CNS involvement.

	Status %(95% CI)	
	CNS involvement	no-CNS involvement
OS			
1-year	90.909 (98.669 - 50.808)	94.118 (98.496 - 78.47)
2-year	79.545 (94.544 - 39.315)	90.98 (97.002 - 74.548)
3-year	79.545 (94.544 - 39.315)	81.834 (92.237 - 60.815)
PFS			
1-year	63.636 (84.516 - 29.689)	80.556 (90.219 - 63.496)
2-year	53.03 (77.315 - 20.933)	80.556 (90.219 - 63.496)
3-year	35.354 (66.787 - 6.869)	75.185 (87.227 - 55.139)

### Adverse effects of combination therapy

Studies show that 70%-90% of patients treated with CAR-T cells experience some degree of CRS, and about 25% develop severe CRS (24, 25). In our study, 43 patients (91.49%) developed CRS, of which 26 (55.32%), 7 (14.89%) and 1 (2.13%) patients experienced Grade 1, 3 and 4 CRS respectively. Despite the high incidence of CRS and no significant advantage compared with other treatment options, the patients displayed good prognosis (**Table 4**). Immune effector cell-associated neurotoxic syndrome (ICANS) is another side effect of CAR-T cell therapy, and although it usually occurs after CRS, some patients develop ICANS even in the absence of CRS. In our study, only 2 (4.2%) patients experienced ICANS, including one with Grade 1 and the other with Grade 4 ICANS (**Table 3**). Furthermore, 20 (42.6%) patients had Grade 2 or lower lymphocytopenia and all patients had Grade 3 or lower hemoglobinemia after transplantation. By day 7 post-transplantation, hemoglobinemia had reduced to Grade 2 or lower in 18 (38.3%) patients, and thrombocytopenia, leukopenia and neutropenia had declined below Grade 2 in 35 (74.4%), 27 (57.4%), and 28 (59.6%) patients respectively by day 14 (**Table 4**). The non-hematological AEs have been summarized in **Table 4**. The most common therapy-associated AEs were infection and infestation. While 31 (64.6%) patients had infection-related complications alone or in combination, 14 (29.79%) patients developed lung infection after the treatment. On the other hand, only 2 (4.17%) patients had treatment-related diarrhea. The incidence rates of intestinal infections and oral ulcers were 39.58% and 27.08% respectively. The less frequent non-hematological AEs included pseudomembranous enteritis, left uveitis, dysregulation of intestinal flora, upper respiratory infection, urinary tract infection, perianal infection, septic shock, and epilepsy (**Table 5**).

**Table 4:** AEs related to CAR-T cell therapy (n=47).

	Grade 0	Grade 1	Grade 2	Grade 3	Grade 4	Total
CRS	4 (8.51%)	26 (55.32%)	9 (19.15%)	7 (14.89%)	1 (2.13%)	43 (91.49%)
ICANS	45 (95.74%)	1 (2.13%)	0 (0%)	0 (0%)	1 (2.13%)	2 (4.2%)

CRS, cytokine release syndrome; ICANS, immune effector cell-associated neurotoxicity syndrome.

**Table 5:** AEs of combination therapy cohorts (n=47).

Hematologic events (Day 7)	Grade 0-2	Grade 3-4
Lymphocytopenia (Day 7)	20 (42.6%)	27 (57.4%)
Thrombocytopenia (Day 14)	35 (74.4%)	12 (25.5%)
Leukopenia (Day 14)	27 (57.4%)	20 (42.5%)
Neutropenia (Day 14)	28 (59.6%)	19 (40.4%)
Hemoglobinemia (Day 7)	18 (38.3%)	29 (61.7%)
Non-hematologic events		
Gastrointestinal disorders		
Gastrointestinal bleeding	5 (10.64%)	
Diarrhea	2 (4.26%)	
Pseudomembranous enteritis	1 (2.13%)	
Left uveitis	1 (2.13%)	
Dysregulation of intestinal flora	1 (2.13%)	
Infection and infestation		
Intestinal infection	19 (40.43%)	
Lung infection	14 (29.79%)	
Upper respiratory infection	1 (2.13%)	
Urinary tract infection	1 (2.13%)	
Perianal infection	3 (6.38%)	
Oral ulcer	13 (27.66%)	
septic shock	1 (2.13%)	
Nervous system disorder		
Epilepsy	1 (2.13%)	

## Discussion

In this study, we evaluated the outcomes of sequential ASCT and CAR-T cell infusion as a salvage therapy for patients with R/R B-NHL. In a previous study, the 1-year OS and PFS rates of R/R B-NHL patients who underwent ASCT after 86.7% and 73.7% respectively, and that in patients who only received CAR-T cell infusion were 79.1% and 55.7% (26). In addition, the 2-year OS and PFS rates of patients treated with the combination of ASCT and CAR-T cell infusion were 78.9% and 66.2% respectively. In our study, the combination therapy extended the 1-year OS and PFS to 89.5% and 75% respectively, and also offered long-term survival benefits, with 3-year OS and PFS reaching 77.1% and 68.8% respectively. Furthermore, our results are more robust since we included patients with different subtypes of B-cell NHL who achieved CR/PR before the combination therapy, and followed up the patients for longer duration. Thus, the combination of ASCT and CAR-T cell therapy offers a new strategy for patients who relapse after first-line therapy.

Although patients with R/R B-NHL can achieve CR/PR after chemotherapy or autologous transplantation, the risk of recurrence is high due to the presence of chemo-resistant residual lesions. Thus, consolidation therapy combining ASCT with CAR-T cells can reduce the risk of recurrence (27). Infusion of autologous HSCs after high-dose chemotherapy or radiotherapy restores the function of the immune system and revives the patient’s T cells (28). In addition, ASCT creates favorable conditions for CAR-T cell activation by mitigating the immunosuppressive cues in the tumor microenvironment, such as immune checkpoint inhibitors, tumor-associated macrophages, etc., thereby improving the therapeutic effect of CAR-T cells. Furthermore, the CAR-T cells not only kill the tumor cells during treatment, but also perform immunosurveillance after treatment to remove the residual tumor cells. Studies show that CAR-T cells can survive for a long time in some patients as immune memory cells, and can therefore respond quickly in the event of relapse (29). Although ASCT can improve the tumor immune microenvironment, the immune escape mechanisms employed by the tumor cells and the resident immunosuppressive components may affect the long-term effects of CAR-T cells. In particular, immunosuppressive cells such as regulatory T cells (Tregs) and myeloid-derived suppressor cells (MDSCs), immune checkpoint molecules like PD-L1, and immunosuppressive cytokines such as TGF-β may limit the activity of CAR-T cells, resulting in variable efficacy (30). In our study, we only selected patients who underwent CAR-T cell re-infusion within two days of ASCT. The number of CAR-T cells peaks within 7-10 days after transplantation, and the hematological recovery post-ASCT usually takes 11 days. Therefore, the two-day interval between the two procedures can minimize the adverse impact of CRS on patients at the peak of CAR-T amplification, and reduce the risk of potential complications. In addition, since the patient’s immune system undergoes a rebuilding process after ASCT, transfusion of CAR-T cells within 2 days can maximize their therapeutic effects before the host immune system fully recovers (31).

We did not observe any unexpected toxicities when administering combination therapy. This can be attributed to the lower tumor burden and better health status of the patients before the combination therapy. CRS is the most common side effect of CAR-T cell immunotherapy, with previously reported incidence rates of 94% (32). In this study, 43 patients (91.49%) developed CRS after CAR-T cell infusion, of which only 8 experienced Grade 3 or higher CRS. In addition, prior infusion of HSCs did not increase the severity of CRS. Approximately 25% of the patients develop ICANS after receiving CAR-T cell therapy (25, 33). The incidence of ICANS in our cohort was only 4.2%, indicating that combination therapy did not increase neurotoxic effects compared to consolidation therapy alone. Furthermore, CAR-T cell infusion did not prolong hematological recovery after ASCT, resulting in low infection rates. Previous studies have shown that median time of neutrophil engraftment after stem cell transplantation is 11 days and that of platelet engraftment is 12 days (34, 36). In our study, the median time of neutrophil and platelet engraftment after combination therapy was 15 days; however, the longer duration did not affect treatment outcomes. The most common non-hematological AEs associated with stem cell transplantation are cytopenia (100%), gastrointestinal toxicity and infection (88.8%), whereas patients treated with CAR-T cells have a lower risk of infection (13.8%) (37). The incidence of lung infection was higher in our cohort compared to that previously reported for ASCT and CAR-T cell monotherapy, but was better controlled. Other common AEs observed after the combination treatment were intestinal infection and oral ulcer, which did have any significant impact on the survival of patients.

Shadman et al. showed that ASCT achieved superior OS and 2-year PFS rates in patients with R/R DLBL compared to CAR-T cell therapy (26). On the other hand, Caixia et al. had demonstrated superior CR and 1-year OS rates, along with significantly lower incidence of severe non-hematological AEs, after CAR- T cell therapy (38). Another study had shown that the combination of CAR-T cell therapy with ASCT improved long-term outcomes in patients with TP53 mutations (39). While this result is consistent with our findings, the disease types reported in the previous study were relatively simple. Nevertheless, the outcomes of the combination therapy were relatively uniform across the different subtypes of B-NHL, which is favorable for clinical application. In addition, there are reports on using CAR-T therapy for tumor reduction prior to ASCT. Preconditioning with CAR-T cells can eradicate tumor cells with reduced toxicity compared to chemotherapy, which is particularly significant for the chemo-resistant patients (40). Despite differences in therapeutic principle, the favorable treatment outcomes of both combination approaches are promising. In line with our results, a recent study showed that the combination of ASCT and CAR-T cell therapy resulted in sustained CR in patients with relapsed/refractory (R/R) Burkitt lymphoma (BL) over a period of 306 days, without any severe complications (41).

There are still several limitations in this treatment approach. Our single-arm design can lead to selection bias, and we set criteria such as specific age ranges, disease types, and treatment response. This may lead to the exclusion of certain patient groups, such as elderly patients or patients with other conditions, affecting the generality of the results. The exclusion of patients who failed to achieve CR or PR prior to CAR-T cell therapy may result in our failure to evaluate efficacy in the context of different treatment responses. Due to the lack of a control group, we cannot make direct comparisons with other treatment options, which can lead to overestimates or underestimates of efficacy.

In future studies, a prospective randomized controlled trial will be designed to compare the efficacy of ASCT combined with CAR-T versus therapy alone. This will help ensure randomness and representativeness of the sample, thereby reducing selection bias.

Future research could focus on personalizing treatment regimen-based on a patient’s genomic profile and tumor microenvironment, exploring the possibility of combining CAR-T cell therapy with other immunotherapies or targeted therapies to enhance therapeutic efficacy and overcome drug resistance. Research can focus on identifying biomarkers associated with treatment response and prognosis, such as specific cytokines, tumor-associated antigens, or circulating tumor DNA (ctDNA). These markers can help predict a patient’s response to treatment and guide clinical decision making.

## Conclusion

The combination of ASCT and CAR-T cell therapy significantly improved the PFS of patients of R/R B-NHL, without any notable increase in toxicity compared to either monotherapy. This combination therapy may be an effective means to improve the long-term survival rates of patients with R/R B-NHL.

## Data Availability

The raw data supporting the conclusions of this article will be made available by the authors, without undue reservation.
